# Preoperative brain shift is a prognostic factor for survival in certain neurosurgical diseases other than severe head injury: a case series and literature review

**DOI:** 10.1007/s10143-021-01659-2

**Published:** 2021-10-07

**Authors:** Paolo Missori, Giuseppe La Torre, Susanna Lazzari, Sergio Paolini, Simone Peschillo, Stefano Martini, Valeria Palmarini

**Affiliations:** 1 Department of Human Neurosciences, Neurosurgery, Policlinico Umberto I, Sapienza” University of Rome, Viale del Policlinico, 155, 00161 Rome, Italy; 2 Department of Public Health and Infectious Diseases, “Sapienza” University of Rome, Rome, Italy; 3 IRCCS Neuromed-Pozzilli, “Sapienza” University of Rome, Rome, Italy; 4 Department of Neurosurgery, University of Catania, Sicily, Italy; 5 Department of Human Neurosciences, Neuroradiology, Policlinico Umberto I, Sapienza” University of Rome, Rome, Italy

**Keywords:** Brain, Head injury, Hemorrhage, Ischemia, Shift, Tumor

## Abstract

Preoperative brain shift after severe brain injury is a prognostic factor for survival. The aim of this study was to determine whether preoperative brain shift in conditions other than severe head injury has significant prognostic value. We analyzed a radiological database of 800 consecutive patients, who underwent neurosurgical treatment. Brain shift was measured at two anatomical landmarks: Monro’s foramina (MF) and the corpus callosum (CC). Four hundred seventy-three patients were included. The disease exerting the highest mean brain shift was acute subdural hematoma (MF 11.6 mm, CC 12.4 mm), followed by intraparenchymal hematoma (MF 10.2 mm, CC 10.3 mm) and malignant ischemia (MF 10.4 mm, CC 10.5 mm). On univariate analysis, brain shift was a significant negative factor for survival in all diseases (*p* < 0.001). Analyzed individually by group, brain shift at both anatomical landmarks had a statistically significant effect on survival in malignant ischemia and at one anatomical landmark in chronic subdural and intraparenchymal hematomas. Multivariate analysis demonstrated that the only independent factor negatively impacting survival was brain shift at MF (OR = 0.89; 95% CI: 0.84–0.95) and CC (OR = 0.90; 95% CI: 0.85–0.96). Brain shift is a prognostic factor for survival in patients with expansive intracranial lesions in certain neurosurgical diseases. MF and CC are reliable anatomical landmarks and should be quoted routinely in radiological reports as well as in neurosurgical practice.

## Introduction

In neurosurgical patients, brain shift on neuroradiological imaging is a key sign in surgical decision-making. The first paper dealing with brain shift in intraparenchymal or subdural hematomas or large middle cerebral artery stroke determined that its progressive increase decreases the level of consciousness, up to deep coma [13]. This result has been confirmed, and a larger mean shift is associated with poor outcomes [14]. The significant value of this measurement to outcomes in severe brain injury has been demonstrated repeatedly [1, 5, 8, 10, 15, 18].

Whether brain shift significantly affects outcomes in other neurosurgical diseases is unclear. Such information could indicate prompt surgical treatment or timely conduct in diseases exhibiting the same brain shift measure. Besides, we daily observe radiological reports lacking brain shift measures or having an overestimated shift missing any anatomic level. This deficiency may either push the neurosurgeon to delay treatment or prompt an emergency procedure, regardless of the patient’s neurological condition. This study aimed to evaluate a standard anatomical level at which to measure brain shift and the prognostic value of brain shift in neurosurgical diseases other than a severe head injury that exert a mass effect, in order to compel the routine inclusion of brain shift measurement in radiological reports and neurosurgical practice.

## Materials and methods

**Fig. 1:**
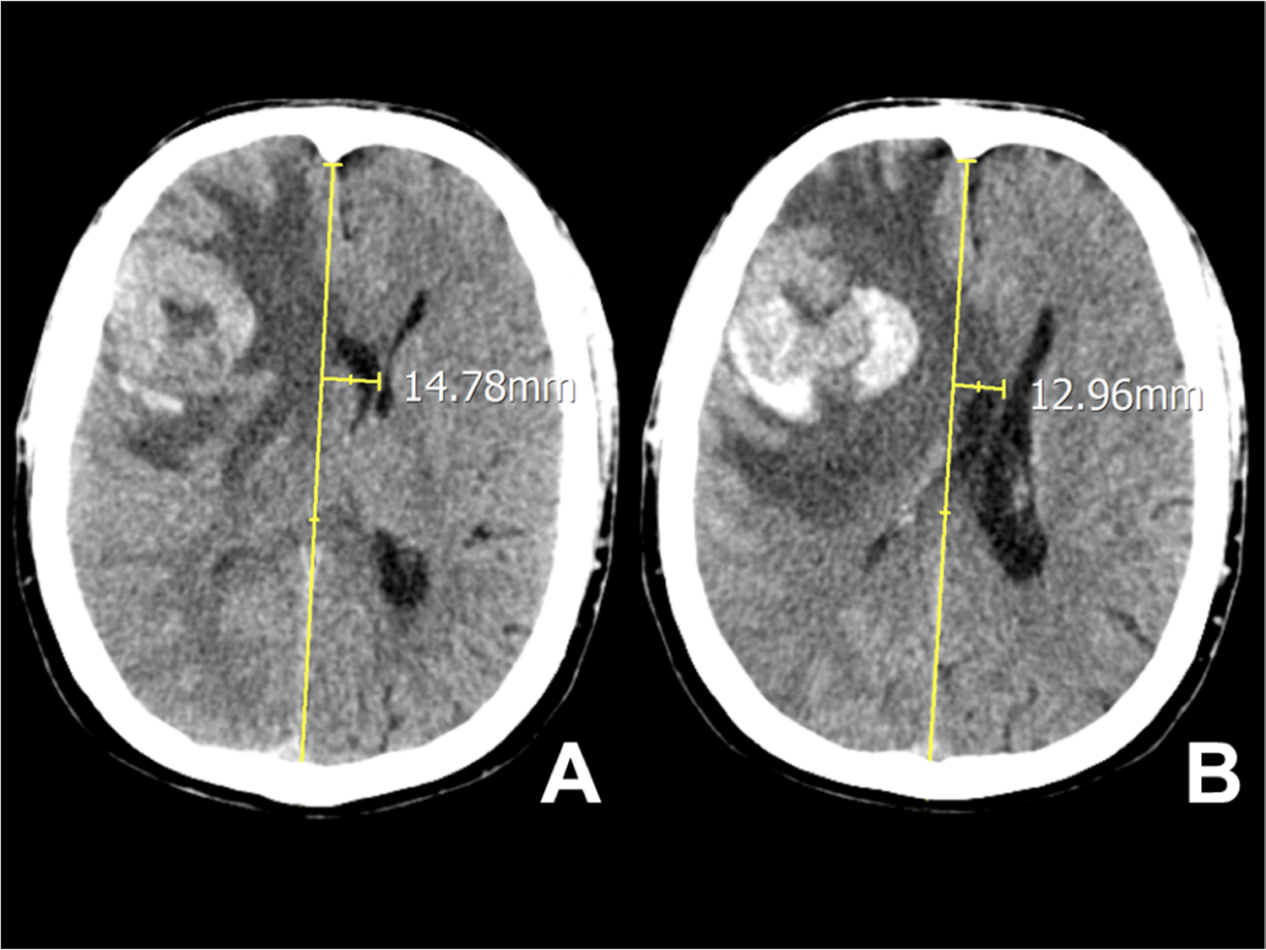
In a patient with a hemorrhagic tumor, brain shift was evaluated in the axial CT scans at the level of MF (**A**). Both frontal horns are displaced from right to left. Using the CT software, a sagittal line (drawn from the crista galli and anterior cerebral falx to the internal occipital protuberance) is intersected by a perpendicular transverse line starting from the septum pellucidum. The midline shift was measured on the transverse line, in millimeters. The same procedure is performed at the CC (**B**)

In this retrospective cohort study, we recruited head computed tomography (CT) data from patients (aged 1–96 years) who were admitted to our emergency department (ED) over a 13-year period (2005–2018). All CT scans were performed for the onset of neurological symptoms or secondary head injury and selected for a disease exerting a mass effect on the brain parenchyma. Patients with primary head injury were strictly excluded. The included patients’ age and sex were recorded. Two observers (one neuroradiologist, one neurosurgeon) calculated the brain shift in the axial scans at the level of Monro’s foramina (MF) and the corpus callosum (CC: the anatomical region in the middle portion of the corpus callosum, corresponding to the body), just above the septum pellucidum and below the cingulate gyrus. These anatomical landmarks are better identified in the displaced brain and are located at a shorter distance from the uncus, where displacement can cause herniation and brainstem damage. The brain shift in the axial CT scan was measured by drawing a sagittal line from the crista galli and anterior cerebral falx to the internal occipital protuberance and an intersecting transverse perpendicular line at the anatomical landmarks (Fig. 1). Brain shift was measured on the transverse line, in millimeters. Agreement between observers was evaluated using the Kappa statistic. We excluded from analysis all scans in which brain shift was absent or the anatomical landmarks were not detectable. All patients underwent surgical treatment and survival was set at 3 months.

### Statistical analysis

The statistical analysis was carried out using the software SPSS for Windows, release 25.0. Medians and interquartile ranges were calculated, as well as respective frequency distributions for quantitative and qualitative variables. Differences between groups were assessed using the Mann–Whitney test. A bivariate analysis (Spearman correlation coefficient) was carried out for age and shifts. Finally, a logistic regression analysis was conducted using as dependent variables the condition of the patients at 3 months (deceased vs. alive) and as independent variables age (as a continuous variable), gender (females vs. males), and hemorrhagic diseases (yes vs. no). Results are reported as OR and 95% CIs. The threshold for statistical significance was set at *p* < 0.05.

## Results

Four hundred seventy-three patients were included. Preoperative head CT scans showed mass effects exerted by intraparenchymal hemorrhage (62 patients), hemorrhage from a ruptured aneurysm (7 patients), malignant ischemia of the middle cerebral artery (14 patients), supratentorial brain tumors (meningiomas, gliomas, metastases:121 patients), acute subdural hematoma (61 patients), chronic subdural hematoma (182 patients), epidural hematoma (22 patients), and brain abscesses (4 patients) (Table 1).

**Table 1:** The median brain shift (in millimeters) was calculated for each disease group. Follow-up was set at 3 months. *MF*, Monro’s foramina; *CC*, corpus callosum

Diseases	Patients, *N*	MF median shift (mm)			CC median shift (mm)		
		Died	Alive	*p* =	Died	Alive	*p* =
Ruptured aneurysm	7	4.09 (4.0–4.0)	5.09 (3.1–18.1)	0.617	2.06 (2.0–2.0)	3.84 (0–18.1)	1.000
Abscess	4	0	7.7 (5.8–9.6)	-	0	9.3 (6.8–11.7)	-
Epidural hematoma	22	9.21 (4.8–12.2)	6.88 (2.5–21.9)	0.395	10.24 (8.0–14.1)	7.44 (2.6–23.4)	0.106
Intraparenchymal hematoma	62	10.4 (5.4–16.0)	8.6 (4.5–20.0)	0.441	11.67 (4.5–16.1)	8.28 (0–23.4)	0.019
Acute subdural hematoma	61	14.34 (3.3–18.2)	12.23 (2.8–19.0)	0.228	15.06 (4.8–18.8)	13.38 (0.9–21.4)	0.326
Chronic subdural hematoma	182	9.09 (4.6–18.5)	8.60 (1.3–19.4)	0.045	10.96 (5.4–20.0)	10.03 (0–18.7)	0.126
Brain tumor	121	8.6 (3.6–14.9)	7.7 (1.7–17.6)	0.427	9.44 (2.5–15.7)	8.5 (0–21.0)	0.493
Malignant ischemia	14	13.3 (11.4–16.1)	9.04 (4.4–14.6)	0.028	12.23 (9.4–14.5)	8.14 (4.8–12.6)	0.028
*Total patients*	473	11.45 (3.3–18.5)	8.48 (1.3–21.9)	< 0.001	11.69 (2.0–20.0)	9.39 (0–23.4)	< 0.001

**Fig. 2:**
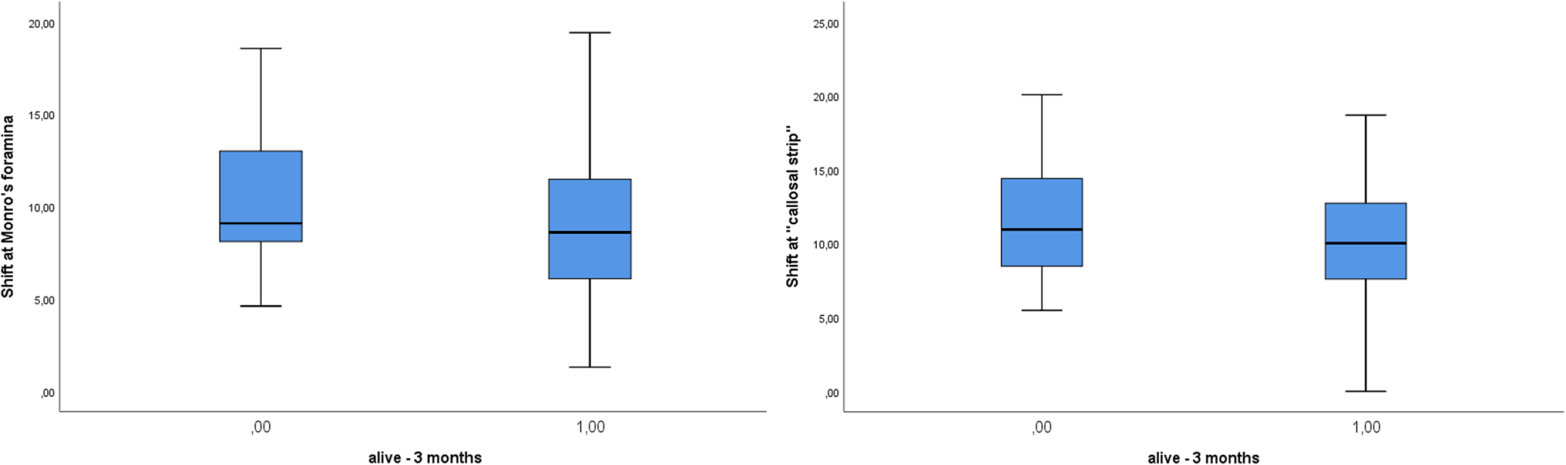
In all patients, mortality increases with increasing shift, at both the MF and CC levels (*p* < 0.001)

The disease with the greatest average shift was acute subdural hematoma (MF 11.6 mm, CC 12.4 mm), followed by malignant ischemia (MF 10.4 mm, CC 10.5 mm), intraparenchymal hematoma (MF 10.2 mm, CC 10.3 mm), and chronic subdural hematoma (MF 9.4 mm, CC 10.3 mm). There were no significant differences between the average values of the shifts at MF and the CC in the different pathologies. The smallest shift was caused by a ruptured aneurysm and abscess. The highest mortalities were found in malignant ischemia (31.2%) and acute subdural hematoma (18.8%). Mortality increased with increasing shift, at both MF and the CC (*p* < 0.001; Fig. 2).

The group analysis shows that brain shift at both anatomical landmarks had a statistically significant effect on survival in malignant ischemia, at MF in chronic subdural hematoma and at the CC in intraparenchymal hematomas. Conversely, brain shift did not affect survival in acute subdural and epidural hematomas, subarachnoid hemorrhage, or tumors. Acute subdural hematoma with the same mean shift as chronic subdural hematoma had 2.4 times greater mortality (*p* < 0.01).

Multivariate analysis demonstrated that the only independent factor negatively impacting survival was brain shift at MF (OR = 0.89; 95% CI: 0.84–0.95) and at the CC (OR = 0.90; 95% CI: 0.85–0.96). The shift was not related to the patient’s age (MF: rho = 0.002; *p* = 0.945; CS: rho = 0.012; *p* = 0.738).

## Discussion

The analysis of our results demonstrates that, analyzing all patients as a group, the overall midline shift is significant at both MF and CC (*p* < 0.001). This is a warning for every neurosurgeon dealing with emergency surgery in the presence of a brain shift on a CT scan. Analyzing by subgroups, only some pathologies are statistically significant. This apparently contradictory result may be due to the small sample size in 2 pathological subgroups, the variable size of each lesion, its position in relation to the midline and skull base, the time of growth which can impair brain perfusion into surrounding brain tissue, and the formation of brain edema.

Previous studies on brain shift in various diseases have not enlightened surgical management. In supratentorial brain tumors, midline shift is an independent predictor of brain swelling after the opening of the dura mater and it is an independent prognostic factor influencing survival among patients with glioblastoma multiforme [3, 11]. In our series of supratentorial brain tumors, brain shift did not have a statistically significant relation to survival, probably due to our collecting both benign and malignant tumors. The slow growth of the tumor causes a progressive adaptation of the compressed brain tissue surrounding the lesion and substantially reduces the median shift compared with that seen in the other acute or subacute pathologies. In patients with spontaneous hemorrhagic lesions, brain shift > 5 mm has prognostic significance for survival [7]. In patients with lobar hemorrhage or cerebral infarction, the degree of septum pellucidum displacement and coma on admission were the only significant factors associated with early death following stroke [9]. Likewise, in our group of surgically treated patients with intraparenchymal hematomas, survival was related to the preoperative brain shift measured at the CC. However, since there was no significant relationship between survival and MF shift, this result should be further investigated in a separate study involving a greater number of patients, the location and volume of blood collection, and the comorbidities that are often present in such cases. In malignant middle cerebral artery ischemia, postoperative midline shift < 5 mm at the level of the septum pellucidum significantly decreased the mortality rate and improved outcome [4, 17]. The clinical result in our small group of patients with malignant ischemia is perfectly in line with the existing literature, and the benefit of this surgical procedure in this disease is now indisputable in our daily practice. One study in chronic subdural hematoma correlated preoperative brain shift and level of consciousness, but not prognosis [16]. Other analyses of chronic subdural hematoma showed no significant association between midline shift on preoperative CT scan and outcome [2, 12, 19]. Our study is the first to highlight the finding that brain shift at MF cannot be ignored in chronic subdural hematoma, although the resulting median shift is not among the largest observed. Obviously, as already stated, the patient’s neurological condition at the time of clinical presentation is of paramount importance.

We have observed no statistical significance in epidural hematoma, acute subdural hematoma, and brain tumors. In such conditions, the prognosis is probably conditioned by preexisting comorbidities or postoperative complications (diabetes, cardio-vascular pathologies, anticoagulation usage, seizures, hospital-acquired infections, etc.) not considered in the present study. We believe the lack of statistical significance of brain shift in patients with subarachnoid hemorrhage is attributable to the low number of such patients in the study. The same mean brain shift in acute and chronic subdural hematomas was associated with the worst prognosis in acute subdural hematomas. This result reveals that, regardless of brain shift, a surgical operation may be less urgent in chronic subdural hematomas, at least for those patients with good Markwalder scores [6]. Conversely, the same degree of shift in acute subdural hematoma requires prompt surgical treatment. The statistically significant prognostic value of shift in one anatomical landmark and lack of it in the other was determined exclusively by the worst median shift detected at the examined level. In our opinion, this difference is mainly due to the location and size of the lesion. We found no statistically significant difference in brain shift distance when measured at MF versus at the CC. For this reason, in every neurosurgical disease exerting a mass effect, brain shift must be measured at least at one of these anatomical levels and described in the report. The clinical evaluation of the patient, which is the paramount factor for every surgical decision, will guide the surgeon to the most appropriate choice for the patient.

### Limitations

The design of this study has some limitations. The primary limitation to the generalization of these results is its retrospective nature. The preoperative baseline of our patient population was recorded only for imaging studies. Preoperative anticoagulation or antiplatelet status was not recorded. A small sample size is in 2 pathological subgroups. In order to deliver an immediate, succinct point to the reader, we intentionally avoided analyzing morbidity and neurological recovery as prognostic factors. In the reported pathologies, morbidity and neurological recovery are time dependent and may vary according to the length of follow-up, the histology, the patient’s age, and the researcher who collects the clinical information. Surgical results were dependent on the surgeon’s ability, adding result bias to the study. The mortality rate was set at the 3-month follow-up.

## Conclusion

Brain shift is a common finding when assessing neurosurgical diseases other than the head injury on preoperative neuroradiological imaging. The MF and CC anatomical landmarks are equally useful in measuring brain shift. Brain shift has significant prognostic value in patients with malignant ischemia or with chronic subdural or intraparenchymal hematoma. Brain shift measurements must be routinely quoted in radiological reports, as well in neurosurgical practice, to avoid delay or undue emergency procedures without prior clinical evaluation of patients.

## Data Availability

The data from the hospital archive are available in an excel format.

## References

[1] Akbik OS, Starling RV, Gahramanov S (2019). Mortality and functional outcome in surgically evacuated acute subdural hematoma in elderly patients. World Neurosurg.

[2] Amirjamshidi A, Abouzari M, Eftekhar B (2007). Outcomes and recurrence rates in chronic subdural haematoma. Br J Neurosurg.

[3] Gamburg ES, Regine WF, Patchell RA (2000). The prognostic significance of midline shift at presentation on survival in patients with glioblastoma multiforme. Int J Radiat Oncol Biol Phys.

[4] Jeon SB, Kwon SU, Park JC (2016). Reduction of midline shift following decompressive hemicraniectomy for malignant middle cerebral artery infarction. J Stroke.

[5] Kotwica Z, Brzeziński J (1993). Acute subdural haematoma in adults: an analysis of outcome in comatose patients. Acta Neurochir (Wien).

[6] Markwalder TM, Steinsiepe KF, Rohner M (1981). The course of chronic subdural hematomas after burr-hole craniostomy and closed-system drainage. J Neurosurg.

[7] Masè G, Zorzon M, Biasutti E (1995). Immediate prognosis of primary intracerebral hemorrhage using an easy model for the prediction of survival. Acta Neurol Scand.

[8] Puffer RC, Yue JK, Mesley M (2018). Long-term outcome in traumatic brain injury patients with midline shift: a secondary analysis of the Phase 3 COBRIT clinical trial. J Neurosurg.

[9] Pullicino PM, Alexandrov AV, Shelton JA (1997). Mass effect and death from severe acute stroke. Neurology.

[10] Quattrocchi KB, Prasad P, Willits NH (1991). Quantification of midline shift as a predictor of poor outcome following head injury. Surg Neurol.

[11] Rasmussen M, Bundgaard H, Cold GE (2004). Craniotomy for supratentorial brain tumors: risk factors for brain swelling after opening the dura mater. J Neurosurg.

[12] Ro HW, Park SK, Jang DK (2016). Preoperative predictive factors for surgical and functional outcomes in chronic subdural hematoma. Acta Neurochir (Wien).

[13] Ropper A (1986). Lateral displacement of the brain and level of consciousness in patients with an acute hemispheral mass. N Engl J Med.

[14] Ross DA, Olsen WL, Ross AM (1989). Brain shift, level of consciousness, and restoration of consciousness in patients with acute intracranial hematoma. J Neurosurg.

[15] Servadei F, Nasi MT, Giuliani G (2000). CT prognostic factors in acute subdural haematomas: the value of the ‘worst’ CT scan. Br J Neurosurg.

[16] Sucu HK, Gelal F, Gökmen M (2006). Can midline brain shift be used as a prognostic factor to predict postoperative restoration of consciousness in patients with chronic subdural hematoma?. Surg Neurol.

[17] Tu PH, Liu ZH, Chuang CC (2012). Postoperative midline shift as secondary screening for the long-term outcomes of surgical decompression of malignant middle cerebral artery infarcts. J Clin Neurosci.

[18] Valadka AB, Gopinath SP, Robertson CC (2000). Midline shift after severe head injury: pathophysiologic implications. J Trauma.

[19] van Havenbergh T, van Calenbergh F, Goffin J (1996). Outcome of chronic subdural haematoma: analysis of prognostic factors. Br J Neurosurg.

